# Bupropion Treatment for Stimulant Withdrawal in a Patient With Substance Use Disorder and Unspecified Bipolar Disorder

**DOI:** 10.7759/cureus.37700

**Published:** 2023-04-17

**Authors:** Daanish Y Khan, Bita C Behaeddin, Stepan Uhlyar, Jonathan M Parker

**Affiliations:** 1 Psychiatry and Behavioral Sciences, St. George's University School of Medicine, Miami, USA; 2 Medicine/Surgery, Jackson Memorial Hospital, Miami, USA; 3 Psychiatry, Jackson Memorial Hospital, Miami, USA

**Keywords:** cocaine withdrawal treatment, bupropion with cocaine use, buproprion cocaine withdrawal, buproprion substance abuse, bupropion psychosis

## Abstract

Patients presenting with comorbid stimulant use disorder is a common occurrence in nearly all medical specialties. New clinical strategies to care for patients experiencing stimulant withdrawal should be considered as an effort to improve clinical outcomes. Our patient, a woman in her early 20s with a history of substance use disorder and unspecified bipolar and related disorder, presented with acute psychosis with symptoms including agitation, auditory hallucinations, and delusions in the context of chronic mental illness and cocaine abuse. She was subsequently admitted to the inpatient psychiatry unit. Notable symptoms included mood swings, erratic behavior, anger, and agitation. Mood and psychotic symptoms were treated with olanzapine. She also received medications, including haloperidol, lorazepam, and diphenhydramine, as needed for agitation, which were given as an emergency treat option (ETO) injection. The patient continuously exhibited irritability and endorsed that she was undergoing cocaine withdrawal symptoms, for which she was started on bupropion. Within days of taking this medication, she reported significant improvement in her psychotic and mood symptoms. The patient continued this treatment during the remainder of her stay until the resolution of her symptoms and was discharged with both bupropion and olanzapine to continue while awaiting an outpatient psychiatry appointment in one week.

## Introduction

Stimulant use disorder in the USA has been a problem that has greatly impacted both the medical system and local communities since the late 1970s and early 1980s [1]. Patients presenting with comorbid stimulant use disorder is a common occurrence in nearly all medical specialties [2,3]. Stimulants affect the central nervous system (CNS) by increasing the synaptic monoamine neurotransmitters dopamine, norepinephrine, and serotonin. Both cocaine and amphetamine exert their effects on the presynaptic aspect of neurotransmitters. Cocaine works through a mechanism of inhibiting the reuptake of monoamine neurotransmitters while amphetamines increase their release [3]. While, currently, the mainstay pharmacologic treatment for acute stimulant intoxication are benzodiazepines, there has been no single medication that has shown consistent results in randomized clinical trials. Bupropion is a commonly prescribed antidepressant that has been approved by the Food and Drug Administration for adult major depressive disorder, seasonal affective disorder (SAD), and smoking cessation. Off-label usage of this medication has been implemented in other illnesses such as attention deficit hyperactivity disorder (ADHD), obesity, and depression associated with bipolar disorder [4,5]. While the clear mechanism of action of bupropion is not completely understood, it is known to exert its effects primarily via the inhibition of neuronal uptake of norepinephrine and dopamine. Recently, bupropion has been documented in the usage of treating withdrawal effects of stimulants such as amphetamine and cocaine. The effects of acute stimulant withdrawal in dependent individuals include a plethora of symptoms, including, but not limited to, feelings of severe dysphoria, irritability, anxiety, and paranoia [6]. Informed consent was obtained from the patient for the publication of this case report.

## Case presentation

A female in her early 20s with a past medical history of multiple substance use disorders, including, but not limited to, cocaine use, 3,4-methyl​enedioxy​methamphetamine (MDMA) use, and marijuana use and unspecified bipolar disorder presented with acute psychosis to our inpatient psychiatric unit after being transferred from the crisis emergency department. She was initially brought by ambulance accompanied by police officers after the officers noted she had "bizarre" behavior, as she was walking around naked throughout busy traffic on the street and yelling profanities. On arrival at the crisis emergency department (ED), the patient was unable to hold a full conversation or sit still without exhibiting agitation. Vitals obtained in the ED displayed a blood pressure of 132/83 mmHg, a respiratory rate of 18, and a pulse rate of 112. On further questioning, the patient admitted to recent substance use of cocaine. Laboratory studies of a urinalysis obtained from the patient had returned positive for cocaine metabolites. The patient denied blood labs to be drawn. She endorsed persecutory hallucinations along with paranoid delusions, accusing the medical team of poisoning and kidnapping her. On her second day in our institution, the patient exhibited several symptoms of stimulant withdrawal, including restlessness, mood swings, paranoia, anger, and agitation, as well as disorganized and impulsive thoughts and behaviors such as running around in the unit, flipping chairs, screaming at staff and other patients. She had initially refused medication treatment. She had exhibited anger upon the interview and had walked away to the opposite side of the unit, screaming, when being questioned by medical staff. Due to erratic and aggressive behaviors, her treatment was initially limited to emergency treat option (ETO) injections consisting of 50 mg of diphenhydramine, 5 mg of haloperidol, and 2 mg of lorazepam. Our patient then made informed consent for medications, and she began treatment with olanzapine. Over the next few days, the patient remained irritable, angry, and hostile, but her psychosis was improving. She began to express her desire for discharge and verbalized having an increased craving for cocaine, claiming she was feeling withdrawal effects. The patient was kept in our institution and started on PO bupropion SR 150 mg to be taken twice a day, which she remained on for the duration of her admission.

On day six of bupropion treatment, the patient reported a major improvement in her withdrawal symptoms. The patient endorsed improvement in her mood, energy, stomach pain, and thoughts. On days seven and eight of bupropion, the patient stated that she felt "really good," explaining that in previous hospital admissions, she would have experienced severe cravings during the entirety of her admission. On day 10 of bupropion use, the patient had substantial resolution of her cocaine withdrawal symptoms with significantly decreased cravings. She was cleared for discharge on day 18 of admission. The patient was discharged with PO bupropion, 150 mg to take twice a day, and PO olanzapine 20 mg once a day. She was advised to follow up in one week with outpatient psychiatry.

## Discussion

**Figure 1:**
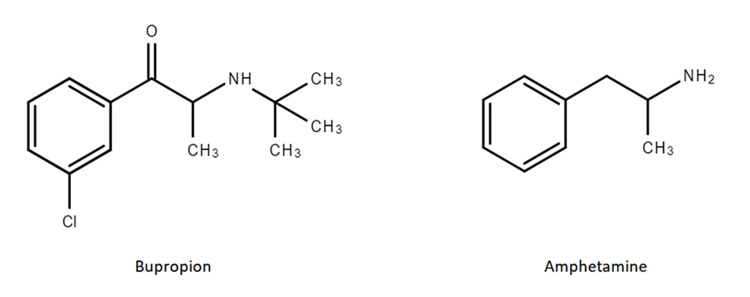
Chemical structures of bupropion and amphetamine

While there is limited data and guidelines in current literature regarding the use of bupropion for the treatment of cocaine withdrawal, there have been few clinical trials that have exhibited the effectiveness of bupropion at reducing cravings, active use, and/or relapse after periods of abstinence [6-9]. The theory behind bupropion's effectiveness stems from the fact that it shares a similar chemical structure to amphetamines, as shown in Figure 1, which allows for cross-reactivity [10].

In 2002, a 12-week clinical trial was executed in order to analyze the treatment of ADHD and cocaine abuse with bupropion. The trial revealed that patients had self-reported a significant decrease in cocaine usage, cocaine cravings, and cocaine-positive toxicologies after receiving daily doses of bupropion in concurrence with weekly individual standardized relapse prevention therapy [8]. Another clinical trial in 2005 assessed the impact of bupropion on the subjective effects of methamphetamine, as well as the impact of bupropion on patients' cravings for methamphetamine. In this trial, patients were given a baseline dose of IV bupropion injection of 0 mg, 15 mg, or 30 mg, followed by a series of methamphetamine IV injections or placebo six days after the bupropion injection. The methamphetamine series was divided into two groups: group A received a 0 mg injection followed by a 30 mg injection, while group B received a 15 mg injection followed by a 30 mg injection. Bupropion treatment was associated with reduced ratings of subjective effects as well as a decrease in general craving using a subjective craving scale. Overall, the data showed that bupropion reduced acute methamphetamine-induced subjective effects and reduced cue-induced cravings in methamphetamine users [9].

A six-month clinical trial in 2006 assessed the impact of bupropion with contingency management on cocaine use reduction. A total of 106 opioid-abusing and cocaine-abusing participants were divided into four treatment groups: contingency management (CM) with placebo, CM with 300 mg/d of bupropion hydrochloride (CMB), voucher control with placebo (VCP), and voucher control with bupropion (VCB). In this study, CM was defined as a type of positive reinforcement, typically in the form of a voucher that was exchangeable for goods or services. A voucher in the study was defined as cash compensation for each submitted urine sample that was negative for cocaine and opioids, as well as cash compensation for participating in weekly abstinence-related activities agreed on during weekly counseling sessions. CMB group participants were given 300 mg/d beginning at week three for the remainder of the study. The study concluded by showing a significant decrease in the proportion of cocaine-positive urine samples in the CMB group from weeks 3-13, relative to week three, and remained low during weeks 14 to 25. In comparison, the CMP group showed a significant increase in cocaine-positive urine samples during weeks 3-13 followed by a decrease in weeks 14-25. Both VCP and VCB groups showed no significant decrease in cocaine-positive urine samples throughout the study [10].

While there is no clear guideline to the use of bupropion in treating and reducing the acute withdrawal effects of stimulants, specifically cocaine, these few clinical trials have shown that the use of bupropion has potential efficacy at reducing cocaine cravings, cocaine use, and reducing relapse use. Prior to starting bupropion therapy in our patient, several important risks were considered in relation to this medication. These risks involve the effects of bupropion on lowering the seizure threshold, the risk of bupropion withdrawal upon abrupt discontinuation of the drug, as well as the possibility of this antidepressant-inducing mania in our patient with a history of bipolar disorder. Firstly, bupropion should be avoided in patients with alcohol use disorder and eating disorders due to its ability to lower the seizure threshold. While this was a big consideration for our patient, our patient's toxicology reports were negative for alcohol at the time of admission, in addition to her denying the use of alcohol upon interview. Our patient also had no documented history of an eating disorder. It was also considered in our patient that bupropion withdrawal may occur, in which follow-up was recommended in order to address this possibility in addition to properly managing her other concurrent medical conditions. As antidepressants are notably known to induce mania in patients with bipolar disorder, it has been noted that bupropion use has been suggested in bipolar patients, especially in the treatment of bipolar depression, due to its low risk of inducing hypomania or mania [11]. After all of these considerations had been carefully addressed, bupropion had been chosen as a promising option in the treatment of our patient.

## Conclusions

Stimulant use disorder is a substance use disorder that affects all aspects of the medical system and is likely to be seen by nearly all physicians at least once in their careers. It is important to remember that not every patient may respond similarly to taking medications to combat their withdrawal symptoms. Bupropion, an amphetamine derivative and commonly used antidepressant, provided a relatively safe and effective treatment option that helped reduce our patient's cocaine cravings and acute withdrawal symptoms throughout her hospital stay. As further research regarding the use of bupropion for stimulant withdrawal continues, promising case studies and new research trials will continue to increase its awareness and effectiveness in being a reasonable option for treating acute stimulant withdrawal.
